# Heparin-Loaded Composite Coatings on Porous Stent from Pure Magnesium for Biomedical Applications

**DOI:** 10.3390/jfb14100519

**Published:** 2023-10-16

**Authors:** Yu-Liang Lai, Cheng-Rui Lin, Chao-Chun Yen, Shiow-Kang Yen

**Affiliations:** 1 Department of Physical Medicine and Rehabilitation, China Medical University Hsinchu Hospital, No. 199, Section 1, Xinglong Road, Hsinchu County 302056, Taiwan; yuliang22@gmail.com; 2 Department of Physical Therapy and School of Medicine, China Medical University, No. 100, Section 1, Jingmao Road, Beitun District, Taichung City 406040, Taiwan; 3 Department of Materials Science and Engineering, National Chung Hsing University, 250, Kuo-Kuang Road, Taichung City 40227, Taiwan; jim04072001@hotmail.com (C.-R.L.); murdocdepp@gmail.com (C.-C.Y.)

**Keywords:** pure magnesium, biodegradable stent, electrolytic load, calcium phosphate, gelatin, heparin, biomedical applications

## Abstract

Challenges associated with drug-releasing stents used in percutaneous transluminal coronary angioplasty (PTCA) encompass allergic reactions, prolonged endothelial dysfunction, and delayed stent clotting. Although absorbable stents made from magnesium alloys seem promising, fast in vivo degradation and poor biocompatibility remain major challenges. In this study, zirconia (ZrO_2_) layers were used as the foundational coat, while calcium phosphate (CaP) served as the surface layer on unalloyed magnesium specimens. Consequently, the corrosion current density was decreased to 3.86, from 13.3 μA/cm^2^. Moreover, a heparin-controlled release mechanism was created by co-depositing CaP, gelatin (Gel), and heparin (Hep) on the specimens coated with CaP/ZrO_2_, thereby boosting magnesium’s blood compatibility and prolonging the heparin-releasing time. Techniques like X-ray diffractometry (XRD), focused ion beam (FIB) system, toluidine blue testing, UV–visible spectrometry, field emission scanning electron microscopy (FESEM), and surrogate tests for endothelial cell viability were employed to examine the heparin-infused coatings. The drug content rose to 484.19 ± 19.26 μg/cm^2^ in multi-layered coatings (CaP-Gel-Hep/CaP-Hep/CaP/ZrO_2_) from 243.56 ± 55.18 μg/cm^2^ in a single layer (CaP-Hep), with the controlled release spanning beyond 28 days. Also, cellular viability assessments indicated enhanced biocompatibility of the coated samples relative to those without coatings. This suggests the potential of magnesium samples after coating ZrO_2_ and CaP with Gel as candidates for porous biodegradable stents or even scaffolds in biomedical applications.

## 1. Introduction

Following the utilization of permanent stents crafted from corrosion-resistant metals for percutaneous transluminal coronary angioplasty, the biodegradable implant is gaining increasing attention for its potential in various medical applications [1,2]. Materials for biodegradable stents must meet several requirements, having good biocompatibility, remaining in situ for several months before they are fully bioresorbed, and the radial force of the resulting scaffold must be sufficient to generate the scaffold effect for the required time [3]. Mg, or magnesium, is a vital ion in the human body, known for its extremely low toxicity, good biodegradability, and outstanding mechanical properties [4]. Based on these requirements, magnesium alloys could be the ideal candidate for this application. This has led to studies focusing on biodegradable metals like Mg and its alloys [5].

In the field of medical research, Ping Lua [6] and his team have made notable contributions by creating PTX-eluting magnesium stents with controllable biodegradability. They accomplished this by using a micro-arc-oxidation/poly-l-lactic acid composite layer on a type of magnesium alloy called AZ81. Nonetheless, there are reservations about magnesium alloys such as AZ91 and AZ81, as they contain percentages of Al. The ions from Al have been linked to Alzheimer’s disease [7]. On a positive note, 2017 saw the announcement of the first clinically proven magnesium scaffold, named Magmaris, by BIOTRONIK. This innovation offers medical practitioners a novel way to treat coronary artery disease, with the advantage of not leaving a permanent implant in the body [8].

Several ceramic coatings have shown promise in reducing abrasion and corrosion when implemented in the human body. Notably, CeO_2_, Ce_2_O_3_, ZrO_2_, Nb_2_O_5_, and MgO have been identified as reliable coatings for protecting magnesium alloys [9]. Among these coatings, zirconia ceramic stands out for its exceptional strength and surface finish, rendering it potentially suitable for various biomedical applications [10]. Furthermore, zirconia coatings, achieved through electrolytic deposition, have demonstrated enhanced corrosion resistance in biological environments on a range of materials, including Co-Cr alloy, 316L stainless steel, Ti-6A1-4V, and AZ91D magnesium alloy [11]. This underscores the potential of zirconia coatings for improving the performance and biocompatibility of these materials in medical settings. In this research, we conduct electrochemical deposition and subsequent annealing of zirconia (ZrO_2_) and calcium phosphate (CaP) coatings on pure magnesium specimens, presenting a promising avenue for the development of biodegradable cardiovascular stents. 

In order to boost magnesium’s blood compatibility and prolong the heparin-releasing time, a controlled heparin release mechanism was created through co-depositing CaP, gelatin (Gel), and heparin (Hep) on the specimens coated with CaP/ZrO_2_ by us. Hydroxyapatite (HAp), characterized by the chemical formula Ca_10_(PO_4_)_6_(OH)_2_, is a primary constituent of human bones. Studies [12,13,14] have shown that when drugs are combined with HAp in coatings on implants via electrochemical methods, they can adopt complex forms and offer continuous drug dispersion as well. This method has been applied to cardiovascular stents and used as a medium in drug delivery systems [15] because of its safe, non-inflammatory, non-blood-breaking, and low clot-promoting properties [16]. 

Thus far, heparin (Hep) remains a favorable clinical option for medical applications. *Heparin*, a highly sulfated glycosaminoglycan, is extensively employed as an injectable anticoagulant due to its remarkably high negative charge density, surpassing that of any other known biological molecule [17]. In a prospective study involving 132 patients with coronary lesions at low risk of restenosis, the use of heparin-coated Wiktor stents in coronary arteries was examined [18]. Gelatin (Gel) is a biocompatible and biodegradable polymer produced through the hydrolysis of collagen. It exhibits zwitterionic properties, containing both carboxyl and amino groups. Additionally, it has been observed that elevating the density of the gelatin membrane can decelerate the release rate of antibiotics [19]. Consequently, Gel represents a promising candidate for interacting with Hep [20], presenting potential opportunities for medical applications.

Recently, Yuebin Lin and colleagues presented a study on heparin/bone morphogenetic protein 2 (BMP2) complexes immobilized on graphene oxide–chitosan (GOCS)–modified magnesium alloy [21]. In their research, they first functionalized graphene oxide (GO) with chitosan, and then they immobilized it on the surface of the magnesium alloy. However, the sustained release of the heparin/BMP2 complex only lasted for 14 days, which limited its potential for clinical application.

In our study, we took a different approach. We performed electrochemical deposition and subsequent annealing to create the foundational coating layer of zirconia (ZrO_2_) and the surface layer, calcium phosphate (CaP), on unalloyed magnesium specimens. This resulted in, as shown in artificial blood plasma at 37 °C by potentiodynamic polarization tests, a significant reduction of the corrosion current density to 3.86 from 13.3 μA/cm^2^. After the addition of Gel, with higher drugs content, the sustained release of the composite lasted for more than 28 days. The extended releasing period and improved corrosion resistance make these coatings a potential advancement in the field of medical applications. 

## 2. Materials and Methods

### 2.1. Materials

In this research, we sourced a magnesium ingot with 99.9% purity from New Chen Te Hang Co., LTD, Kaohsiung City, Taiwan, and shaped it into plates of dimensions 20 × 20 × 1.4 mm^3^, which were used as the metal bases. Table 1 provides a comprehensive chemical breakdown of the magnesium. Before any tests, each sample was polished with SiC paper, having grit sizes between 600 and 1000, procured from Sanpany Co., Taipei City, Taiwan. Post-polishing, the specimens were meticulously cleaned using ethanol within an ultrasonic bath for a span of 10 min and then dried using blown air.

Heparin sodium powders were procured from Sigma-Aldrich (H3149, Grade I-A, ≥180 units/mg), St. Louis, USA, while gelatin (Gel) was acquired from Fluka Chemie, Biochemica 48723 (bloom 160), Buchs, Germany.

### 2.2. Cathodic Polarization Examinations and Accumulation

As per the previous paper, the potentiostatic method has been successfully employed for Zr (OH)_4_ coating on AZ91D magnesium alloy [11]. However, a challenge arises when dealing with pure magnesium, primarily because of its high corrosion rate in Zr O(NO_3_)_2_ acidic solution [22]. Consequently, prior surface pre-treatment is essential to protect the Mg specimen before proceeding with the electrolytic Zr (OH)_4_ deposition.

The procedure began by immersing the substrates in 1 M NaOH for 24 h to create Mg (OH)_2_ films, thereby leading to spontaneous passivation of the substrates [23]. Next, Zr (OH)_4_ films were deposited on plates, which served as working electrodes, using a platinum foil as the counter electrode and a saturated Ag/AgCl reference electrode. This deposition process was carried out in 0.005 M Zr O(NO_3_)_2_ solutions using an EG & G VersaStat™ II along with Power Suite software 352 (Princeton, NJ, USA). Afterward, the coated specimens were left to dry overnight and then annealed in air at 350 °C for 1 h.

Following the above steps, the second layer of HA was coated onto the Mg specimen previously coated with ZrO_2_. The HA coating procedure was conducted at 65 °C in a combined solution of 0.042 M Ca (NO_3_)_2_.4H_2_O (SHOWA, Tokyo, Japan) and 0.025 M NH_4_H_2_PO_4_ (SHOWA, Japan), creating the specified CaP solution.

The application of the third layer, calcium-phosphate–heparin (CaP-Hep) composite, occurred in a CaP solution combined with 800 IU/mL heparin, known as the CaP-Hep solution.

Subsequently, for the fourth layer, calcium-phosphate–gelatin–heparin (CaP-Gel-Hep) composite, the deposition occurred in a CaP solution containing 1 wt.% gelatin and 800 IU/mL heparin, designated as the CaP-Gel-Hep solution.

**Figure 1:**
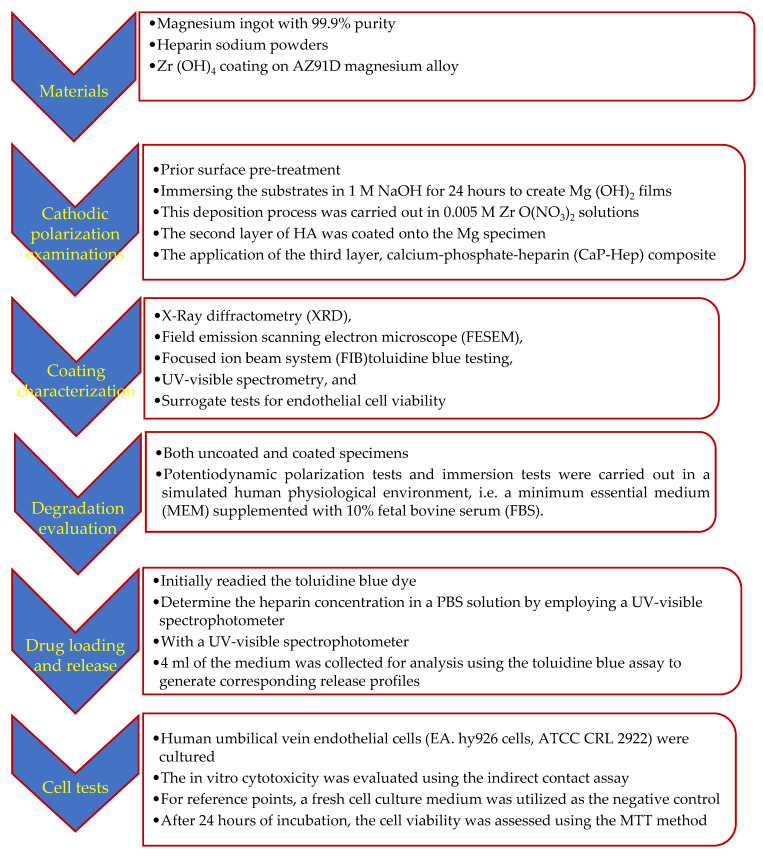
The experimental procedures summary.

Figure 1 show the procedures summary of this study. 

To explore the deposition mechanism, we subjected both uncoated and coated specimens to dynamic polarization in various solutions, including CaP-Gel-Hep, CaP-Hep, CaP, Hep (800 IU/mL), and DI water. The pH values (measured with a pH meter—CYBERSCAN-500, Blue Ash, OH, USA), O_2_ concentrations (measured using an O_2_ meter), and conductivities (measured with a conductivity/°C meter—COSCAN CON6, West Tasman Drive San Jose, CA, USA) of these electrolytes were recorded and are provided in Table 2.

### 2.3. Coating Characterization

The crystal structures of the coated specimens were analyzed using X-ray diffractometry (XRD) with a BRUKERS MXP-III instrument from Germany. This XRD device utilized a 2.0 kW Cu Kα radiation source (λ = 1.5418 Å), operating at 40 kV and 30 mA. The input grazing angle was always set to 1°, with a scan rate of 2°/min, covering a range from 10° to 70°. For detailed examination of the layered configuration and depth profiles of the composite coatings, a focused ion beam (FIB) system (JIB4601F, JEOL, Tokyo, Japan) equipped with energy-dispersive spectroscopy was used.

### 2.4. Degradation Evaluation

To evaluate the biodegradation (corrosion resistance) of uncoated and coated specimens, potentiodynamic polarization tests and immersion tests were carried out in a simulated human physiological environment, i.e., a minimum essential medium (MEM) supplemented with 10% fetal bovine serum (FBS) [24]. 

A medium enriched with 10% FBS in MEM offers a more accurate in vitro representation compared to other simulated body fluids (SBFs) because, much like human plasma, it comprises not just inorganic ions but also organic molecules including amino acids and proteins [25,26]. Concentrations of major components in blood plasma and MEM supplemented with 10% FBS are shown in Table 3. 

Potentiodynamic polarization tests took place in a cell holding 300 mL of electrolyte at 37 °C with a 0.5 mV/s scanning rate. Both uncoated and coated Mg samples, acting as the working electrode, had an area of 1 cm^2^ exposed to the electrolyte. For a closer mimicry of degradation and corrosion patterns found within the human body, we conducted immersion assessments in sterilized containers holding 27.5 mL of MEM enriched with 10% FBS at 37 °C under a humidified 5% CO_2_ environment. Every day, 15 mL of the medium was replenished to emulate the typical 2.75 L plasma volume in adults and the 1.5 L urinary output [27]. Following various immersion durations, the samples were taken out of MEM, carefully washed with deionized water, and dried at 55 °C using a drying device for subsequent examinations. The specimens’ microstructural and compositional alterations were identified using FESEM, while GAXRD was used to determine crystallinity.

### 2.5. Drug Loading and Release

Before commencing drug loading or in vitro release tests [28], we initially readied the toluidine blue dye. This involved dissolving 25 mg of toluidine blue in a 500 mL solution containing 0.2% NaCl and 0.01 N HCl. The resulting solution was then used to determine the heparin concentration in a PBS solution by employing a UV–visible spectrophotometer. To create phosphate buffer solution (PBS-A), we dissolved 8 g NaCl, 0.2 g KCl, 1.11 g Na_2_HPO_4_, and 0.2 g KH_2_PO_4_ in 1 L of distilled water. 

After acidifying the solution with 0.01 M HCl, we prepared the heparin-loaded coatings by dissolving them in 4 mL of PBS-A and blending with 4 mL of toluidine blue for 2 h, creating a heparin–dye complex. We then added 3 mL of n-hexane to each vial and mixed them, resulting in the separation of the solution into two phases: the complex on top and the aqueous segment below. The absorbance of the separated water-based solution, diluted using 99.5% alcohol, was gauged at 628 nm with a UV–visible spectrophotometer (UV–vis; HITACHI U3010, Tokyo, Japan) to identify the heparin concentration. To study the release dynamics of the coatings, heparin-laden samples with a surface area of 1 cm^2^ were submerged in 4 mL of PBS at 37 °C and oscillated at 120 rpm on a water bath shaker (YIHDER BT-150).

At specific intervals, 4 mL of the medium was collected for analysis using the toluidine blue assay to generate corresponding release profiles. Afterward, the collected medium was replaced with 4 mL of fresh PBS for continuous monitoring.

### 2.6. Cell Tests

Human umbilical vein endothelial cells (EA. hy926 cells, ATCC CRL 2922) were cultured in Dulbecco’s modified Eagle’s medium (DMEM) supplemented with 10% fetal bovine serum (FBS). The cells were maintained in a 5% CO_2_ atmosphere at 37 °C. The growth medium was replenished every 3 days until the cells reached confluence. Upon reaching confluence, the cells were detached using a 0.25% trypsin–EDTA solution (GIBCO, Stratford, ON, Canada) and then seeded onto the specimens in a 75-well cell culture cluster (COSTAR, Washington, DC, USA) at a density of 5 × 10^5^ cells/well in fresh medium.

According to ISO 10993-5, the in vitro cytotoxicity was evaluated using the indirect contact assay. Cells were seeded in each well of a 96-well cell culture plate at a density of 5 × 10^3^ cells/100 μL of culture medium and allowed to attach for 24 h [29]. 

Following this, the culture medium was switched out with extracts derived from the immersion test of either uncoated or coated samples under standard conditions (1.25 cm^2^/mL of culture medium at 37 °C for a day). This procedure was designed to mimic clinical usage scenarios and evaluate the potential toxicity of the tested specimens. For reference points, a fresh cell culture medium was utilized as the negative control to set the baseline reaction, while the culture medium enriched with 5% di-methyl-sulfoxide (DMSO) served as the positive control, confirming the test system’s apt response.

After 24 h of incubation, the cell viability was assessed using the MTT method [30]. This method relies on the ability of metabolically active cells to convert a yellow tetrazolium salt (3[4,5-dimethylthiazol-2-y1]-2,5-diphenyltetrazolium bromide) to a formazan product. A 100 μL solution of 0.5 mg/mL MTT was added to each well, followed by a 4-h incubation at 37 °C. The formazan was then dissolved using 100 μL of DMSO with gentle agitation for 10 min. The optical absorbance, measured at 540 nm using a microplate reader (Stat Fax-2100, Awareness Technologies, Chicago, IL, USA), was directly proportional to the number of living cells.

### 2.7. Statistical Analysis

The Student’s *t*-test in the Microsoft Excel statistical program was employed to evaluate the variations in medication encapsulation, weight increase, and cell viability between the groups throughout the study. A *p*-value less than 0.05 indicates statistical significance between the groups.

## 3. Results

### 3.1. Polarization

**Figure 2:**
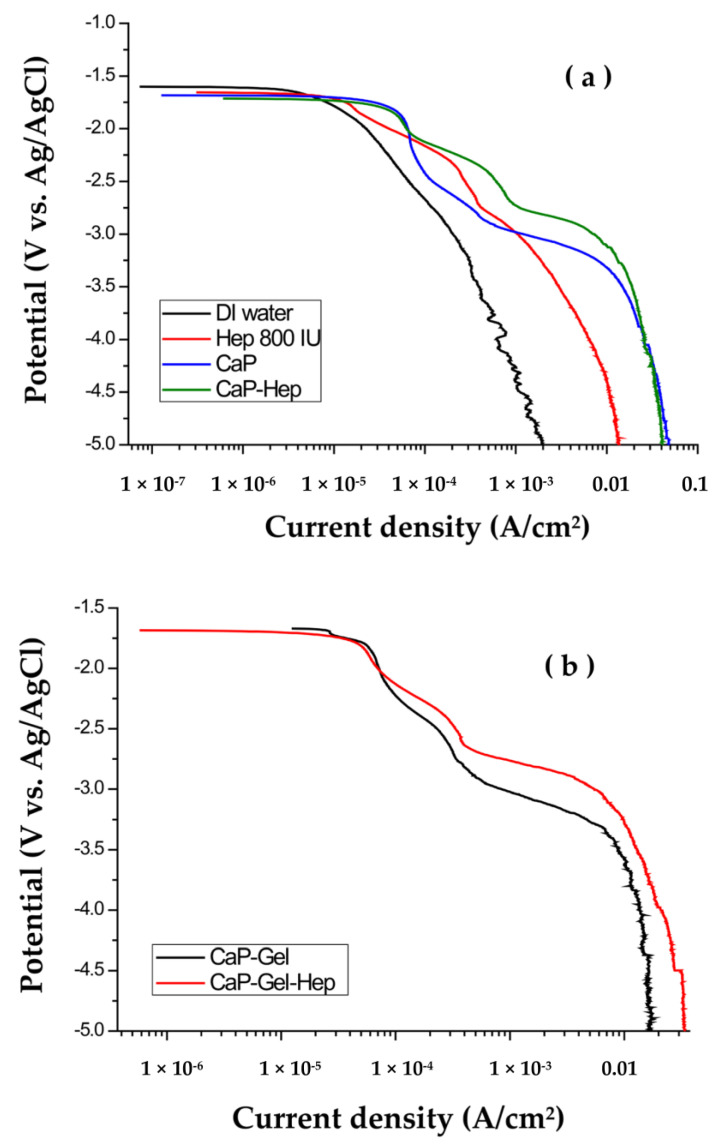
(**a**) Cathodic polarization curves of CaP/ZrO_2_-coated specimens in CaP-Hep solutions, CaP, Hep, and DI water. (**b**) Cathodic polarization curves of CaP-Hep/CaP/ZrO_2_-coated specimens in CaP-Gel-Hep solutions and CaP-Gel.

Figure 2a displays the cathodic polarization curves of CaP/ZrO_2_-coated specimens in various solutions, namely DI water, Hep, CaP, and CaP-Hep. Due to magnesium’s more active redox potential (−2.37 V) compared to that of NiTi alloy (−0.25 V for nickel, −0.86 V for titanium), Mg exhibits a more active open circuit potential (−1.65 V vs. Ag/AgCl) than NiTi alloy (−0.36 V vs. Ag/AgCl).

In the cathodic polarization curve obtained in DI water, no obvious step is observed, indicating a simple reaction involving the electrolysis of water.

2 H_2_O + 2 e^−^ → H_2_ + 2OH^−^ (1)

Upon the addition of heparin (800 IU) to DI water, there is a noticeable rise in conductivity. This increase in conductivity can be attributed to the catalytic reaction of heparin, which accelerates the electrolysis of water. As a result, the current density observed in the cathodic polarization curve is greater in Hep solution compared to DI water.

However, due to the repulsive forces among the negatively charged heparin molecules, the electrodeposition process results in very minimal drug loading on the substrates, making it challenging to measure.

The polarization curves of specimens coated with CaP/ZrO_2_ in CaP and CaP-Hep solutions exhibit striking similarities. These curves can be categorized into three distinct regions, and their corresponding electrochemical reactions were comprehensively elucidated in the previous study [31].

In zone I (−1.7 V~−2.15 V), the reaction is associated with the levels of H^+^ and O_2_ in CaP and CaP-Hep mixtures and can be characterized as follows.

4H^+^ + O_2_ + 4e^−^ → 2H_2_O (2)

H_2_O + O_2_ + 2e^−^ → 2OH^−^ (3)

In zone II (−2.15 V~−2.75 V), with the more negative potential applied, the subsequent reactions take place.

2H_2_PO_4_^−^ + 2e^−^ → 2HPO_4_^2−^ + H_2_ (4)

HPO_4_^2−^ + 2e^−^ → PO_4_^3−^ + H_2_ (5)

H_2_PO_4_^−^ + 2e^−^ → PO_4_^3−^ + H_2_ (6)

From Reactions (4), (5), and (6), some chemical reactions of calcium phosphate occur as follows.

Ca^2+^ + HPO_4_^2−^ + 2H_2_O → CaHPO_4_ · 2H_2_O (DCPD) (7)

8Ca^2+^ + 2 HPO_4_^2−^ + 4PO_4_^3−^ + 5H_2_O → Ca_8_H_2_(PO_4_)_6_ · 5H_2_O (OCP) (8)

In region III (below −2.75 V), there are a lot of hydrogen bubbles, coming from the electrolysis of water in Reaction (1). The massive quantity of OH^−^ in Reaction (10) promotes the formation and deposition of hydroxyapatite on substrates by the following reaction.

10 Ca^2+^ + 6 PO_4_^3−^ + 2 OH^−^ → Ca_10_(PO_4_)_6_(OH)_2_ (9)

Hence, the coatings of CaP-Hep and CaP-Gel-Hep are executed in zone III.

In Figure 2b, we observe the polarization curves of CaP-Hep/CaP/ZrO_2_-coated specimens in both CaP-Gel and CaP-Gel-Hep solutions. Remarkably, these curves bear a striking resemblance to the ones obtained from CaP/ZrO_2_-coated specimens in CaP and CaP-Hep solutions, without the inclusion of Gel.

### 3.2. Characterization of the Coatings

#### 3.2.1. X-ray Diffraction

**Figure 3:**
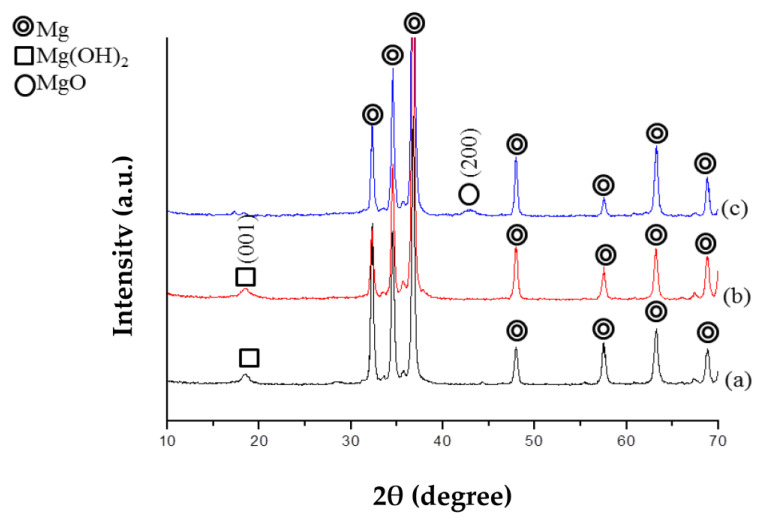
XRD diagrams of pre-treated (a), as-coated (b), and post-annealed ZrO_2_-coated Mg specimens (c).

Figure 3 displays the X-ray diffraction patterns of three types of specimens: pre-treated, as coated, and ZrO_2_ post-annealed coated. On the pre-treated Mg specimen, the presence of the (001) peak of Mg (OH)_2_ film indicates the formation of passivating films resulting from immersion in 1 M NaOH solution.

In contrast, the post-annealed ZrO_2_-coated specimen does not exhibit diffraction peaks of Mg (OH)_2_ or ZrO_2_. This is because the annealing temperature of 350 °C was lower than that required for the crystallization of tetragonal/monoclinic ZrO_2_ [11].

However, the annealing temperature of 350 °C does facilitate the transformation of Mg (OH)_2_ into MgO [32], leading to the detection of the MgO (200) peak on the post-annealed ZrO_2_-coated specimen.

**Figure 4:**
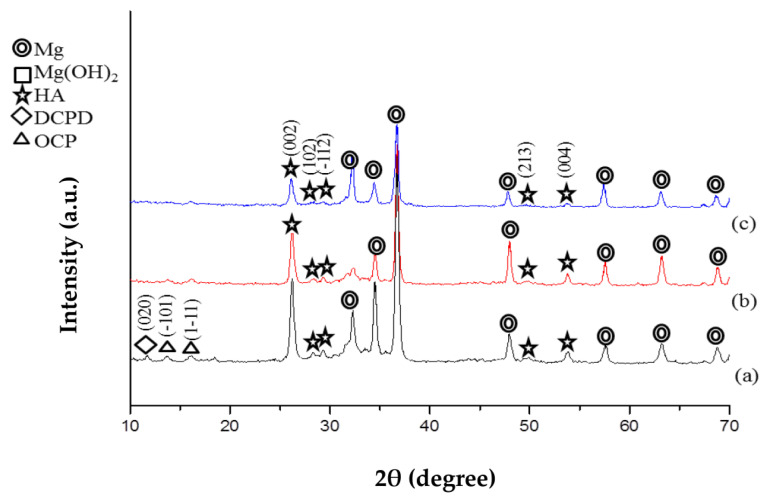
XRD diagrams of CaP coating on untreated (a), pre-treated (b), and ZrO_2_-coated Mg specimens (c).

**Figure 5:**
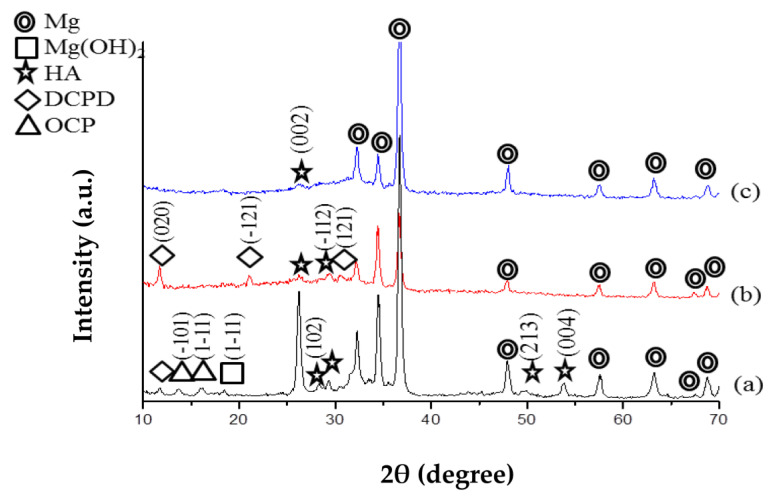
XRD diagrams of CaP-coated (a), CaP-Gel-Hep-coated (b), and CaP-Hep-coated Mg specimens (c).

Figure 4 illustrates the X-ray diffraction (XRD) patterns of CaP coating on three types of Mg specimens: untreated (a), pre-treated (b), and ZrO_2_ coated (c). Similarly, Figure 5 shows the XRD patterns of CaP-Hep (b) and CaP-Gel-Hep (c) coatings on untreated specimens. The coatings are primarily made up of hydroxyapatite (HA, No. 72-1243), a dominant component in calcium phosphates.

In the coatings that lack the ZrO_2_ base layer, both dehydrated di-calcium phosphate (DCPD, No. 72-1240) and octa-calcium phosphate (OCP, No. 79-0423) can be found. Yet, these stages vanish when CaP is applied to the specimen coated with ZrO_2_. This occurs as the electrolytic HA coating procedure necessitates an adequate provision of OH^−^ ions. The presence of ZrO_2_ coating (initially Zr (OH)_4_ before annealing) offers an abundant source of OH^−^ ions, making it easier to form pure HA compared to bare magnesium substrates.

**Figure 6:**
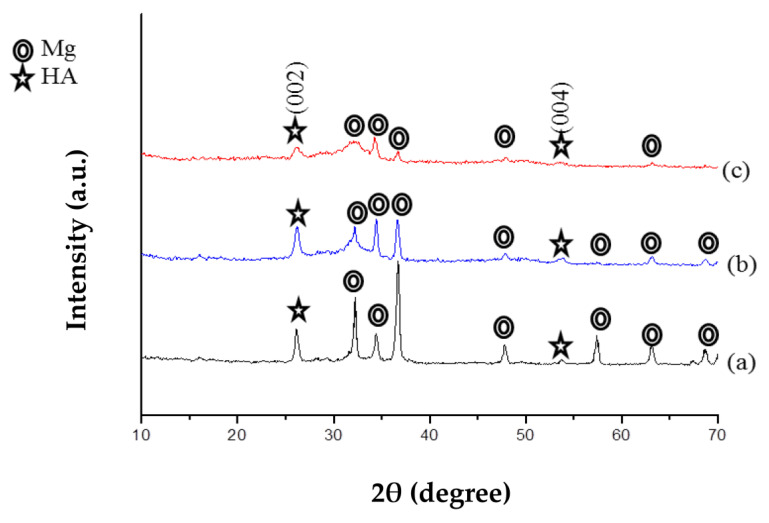
XRD patterns for CaP/ZrO_2_-coated specimens (a), specimens coated with CaP-Hep/CaP/ZrO_2_ (b), and those with CaP-Gel-Hep/CaP-Hep/CaP/ZrO_2_ on Mg (c).

Figure 6 showcases the X-ray diffraction (XRD) patterns for CaP-Hep and CaP-Gel-Hep coatings on CaP/ZrO_2_-coated specimens and CaP-Hep/CaP/ZrO_2_-coated specimens, depicted in (b) and (c), respectively. For both instances, the magnitude of magnesium and HA diminishes, indicating a growth in the coating’s thickness. Additionally, the presence of heparin and/or gelatin interferes with the crystal growth of HA, resulting in broader peaks for HA in the XRD patterns.

**Figure 7:**
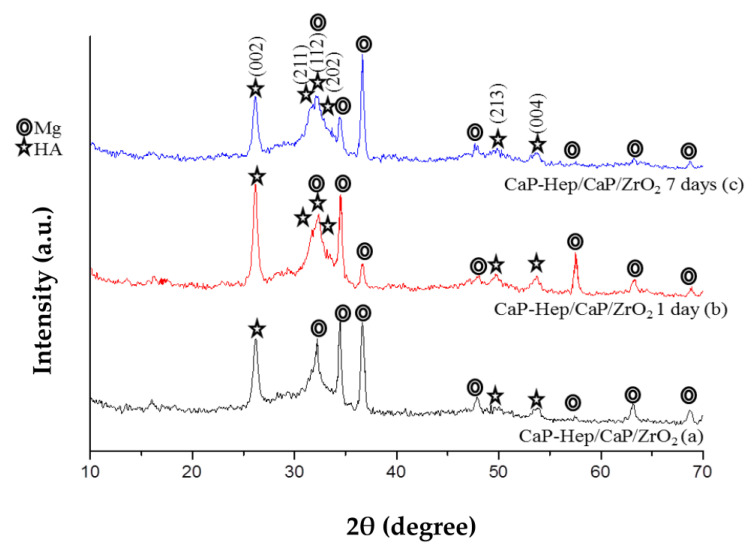
XRD patterns for Mg specimens coated with CaP-Hep/CaP/ZrO_2_ (a) and following immersion release at 1 day (b) and 7 days (c).

In Figure 7, the X-ray diffraction (XRD) patterns reveal that the intensities of HA (211), (112), and (202) planes in CaP-Hep/CaP/ZrO_2_-coated specimens increase with immersion time. This indicates that re-precipitation of HA occurs with a random orientation during the immersion process, which differs from the preferred (200) orientation of HA on specimens achieved through electrodeposition.

**Figure 8:**
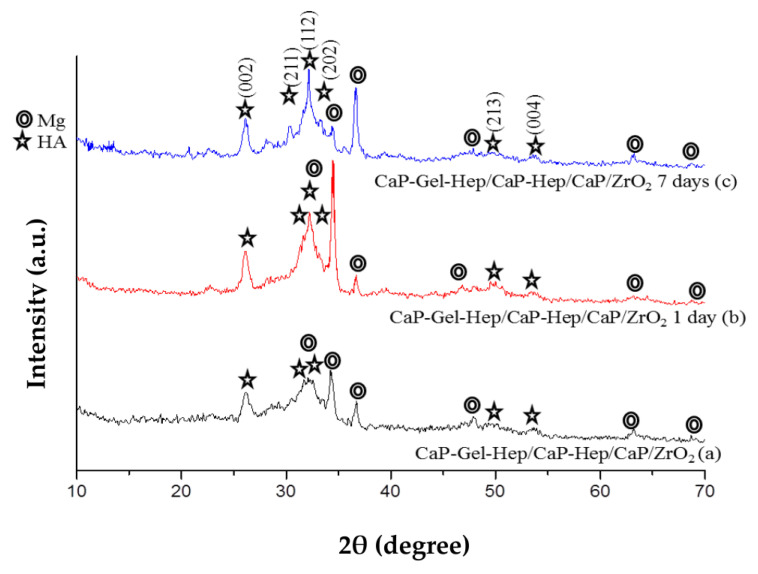
XRD patterns for Mg specimens coated with CaP-Gel-Hep/CaP-Hep/CaP/ZrO_2_ (a), followed by immersion release results at 1 day (b) and 7 days (c).

Similarly, in Figure 8a–c, the XRD patterns of specimens coated with CaP-Gel-Hep/CaP-Hep/CaP/ZrO_2_ also exhibit results consistent with random orientation, further supporting the occurrence of re-precipitation during the immersion process.

#### 3.2.2. FESEM

**Figure 9:**
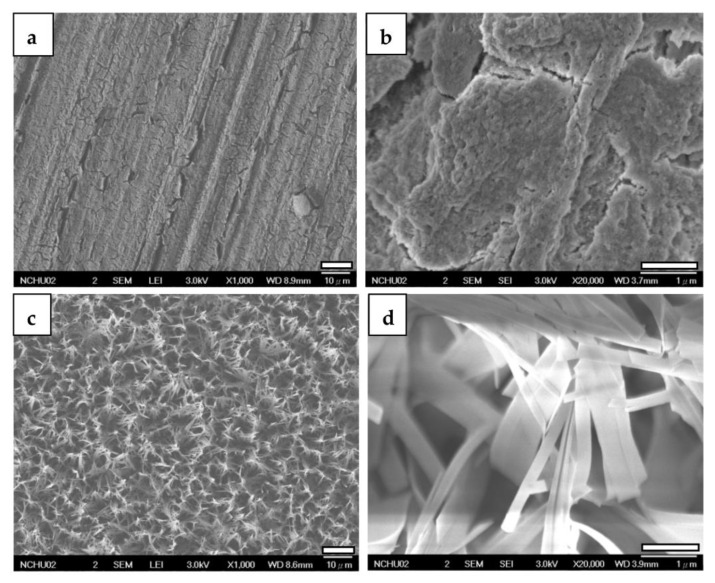
FESEM images of ZrO_2_-coated (**a**,**b**) and CaP/ZrO_2_-coated (**c**,**d**) Mg specimens.

Figure 9 displays FESEM images of specimens coated with ZrO_2_ and CaP/ZrO_2_. In the higher magnification, the ZrO_2_ coating exhibits mud cracks and polished scars clearly resulting from mechanical grinding. However, on the CaP/ZrO_2_-coated specimens, a grass-like HA layer is uniformly deposited at 65 °C, revealing a porous and crack-free surface.

**Figure 10:**
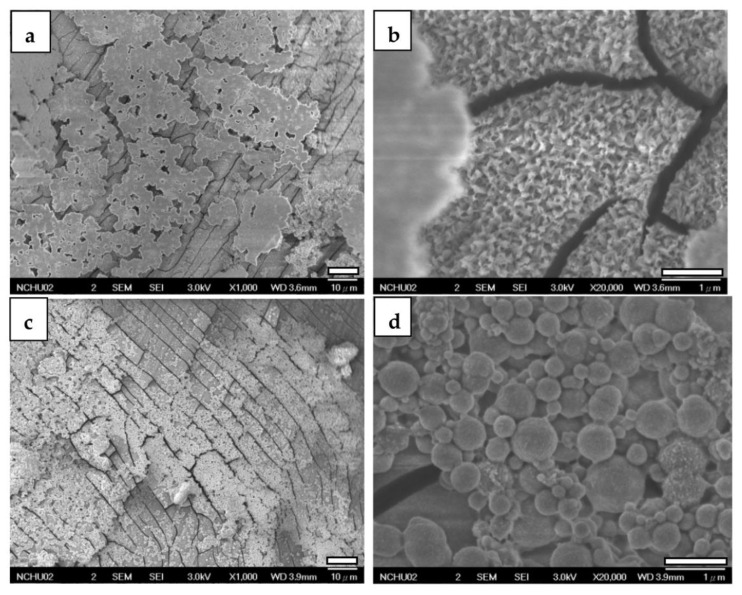
FESEM photographs of the CaP-Hep-covered (**a**,**b**) and the CaP-Gel-Hep-covered (**c**,**d**) Mg samples.

When examining CaP-Hep and CaP-Gel-Hep coatings without post-annealed CaP/ZrO_2_ coating, a consistent two-layer structure is observed, with distinct morphologies and cracks detected on the under layer, as depicted in Figure 10a,b and Figure 10c,d. The former exhibits irregular cloud-like films on the top layer, while the latter shows microspheres on its top layer.

**Figure 11:**
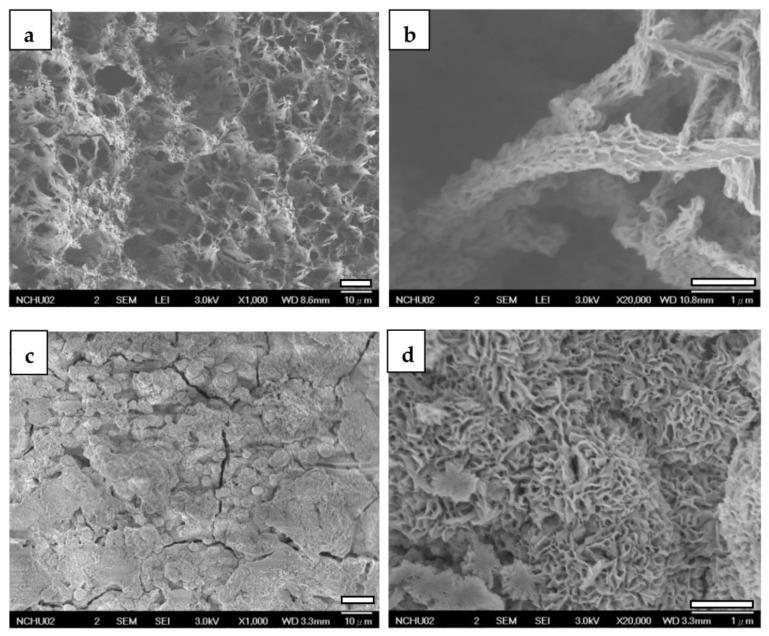
FESEM images of CaP-Hep/CaP/ZrO_2_-covered (**a**,**b**) and CaP-Gel-Hep/CaP-Hep/CaP/ZrO_2_-covered (**c**,**d**) Mg samples.

Upon depositing CaP-Hep on CaP/ZrO_2_-coated specimens, more fine-needle-like particles grow on the grass-like HA of the lower layer, maintaining the porous structure, as illustrated in Figure 11a,b.

Conversely, the fourth layer of CaP-Gel-Hep fills the pores roughly, forming a globular structure with a denser appearance and some cracks. These cracks may result from the dehydration of the gelatin-containing layer, leading to shrinkage, as shown in Figure 11c,d.

**Figure 12:**
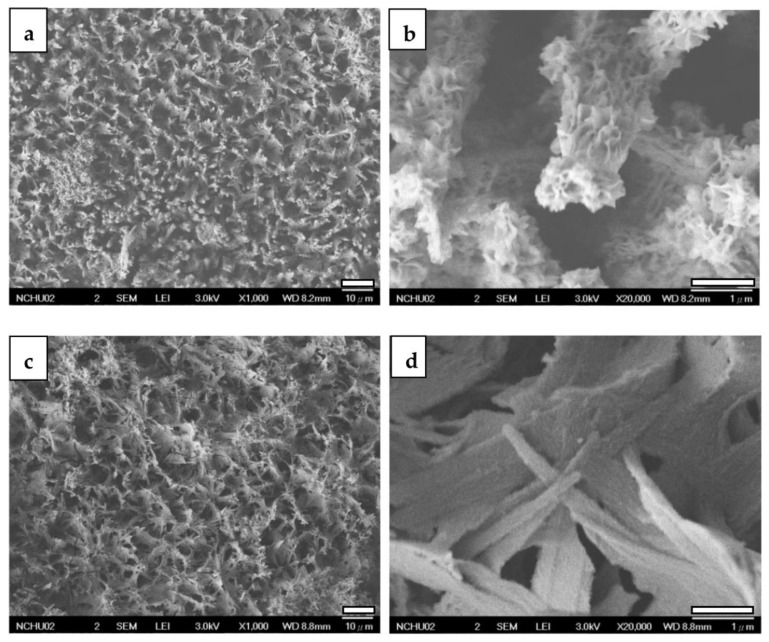
FESEM images of CaP-Hep/CaP/ZrO_2_-covered Mg samples after 1-day drug release (**a**,**b**) and 7 days (**c**,**d**).

Figure 12a,b showcases FESEM views of CaP-Hep/CaP/ZrO_2_-coated specimens after a 1-day immersion in PBS solution. These images reveal a coral-like structure with a rough and porous surface, corresponding to the random orientation of re-precipitated HA shown in Figure 7b. However, after 7 days of immersion, as shown in Figure 12c,d, the images display porous denser leaf-like flakes. This transformation is attributed to drug release, gelatin degradation, and the re-precipitation of HA, resulting in a different structure compared to the grass-like CaP/ZrO_2_-coated specimens shown in Figure 9c,d.

**Figure 13:**
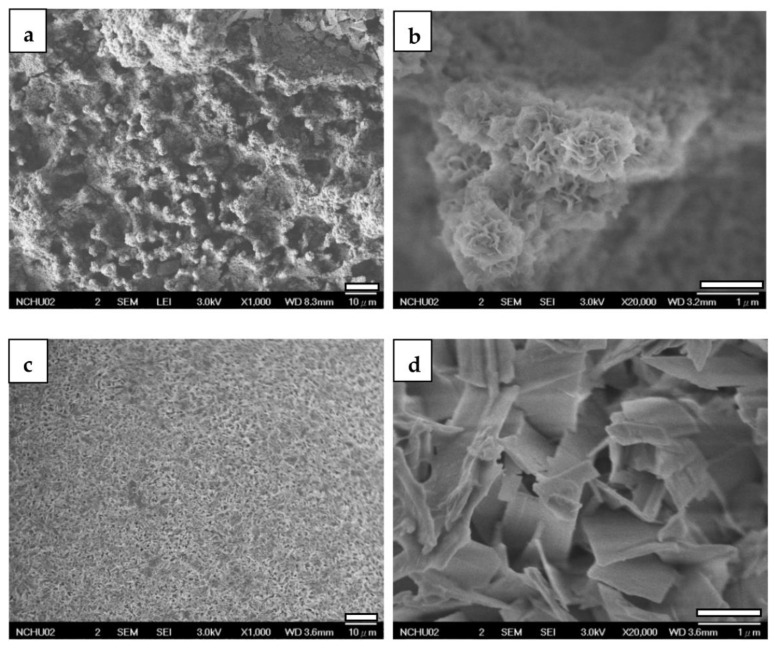
FESEM images of CaP-Gel-Hep/CaP-Hep/CaP/ZrO_2_-covered Mg samples after drug release for 1 day (**a**,**b**) and 7 days (**c**,**d**).

After 1 day of immersion, the CaP-Gel-Hep/CaP-Hep/CaP/ZrO_2_-coated specimen exhibits rounder rose-like particles on the top layer. However, after 7 days of immersion, the top layer of the CaP-Gel-Hep coating is nearly dissolved, leaving behind a layer composed of dense, thick, small, and rectangular particles. This transformation is a result of the dissolution of the top two layers and then the re-precipitation of HA, as illustrated in Figure 13c,d.

#### 3.2.3. Cross-Section Observations by Focused Ion Beam (FIB) System 

**Figure 14:**
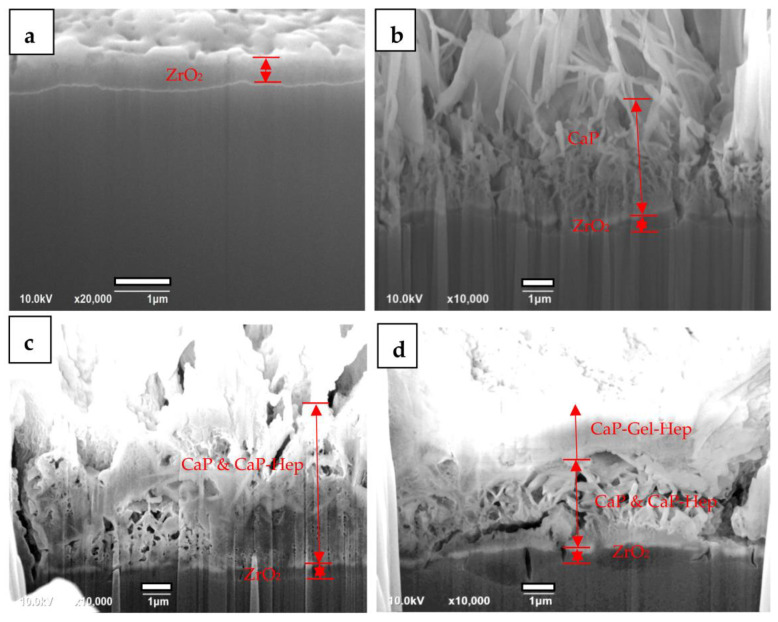
FIB sectional view of Mg specimens with ZrO_2_ coating (**a**), CaP/ZrO_2_ coating (**b**), CaP-Hep/CaP/ZrO_2_ coating (**c**), and CaP-Gel-Hep/CaP-Hep/CaP/ZrO_2_ coating (**d**).

**Figure 15:**
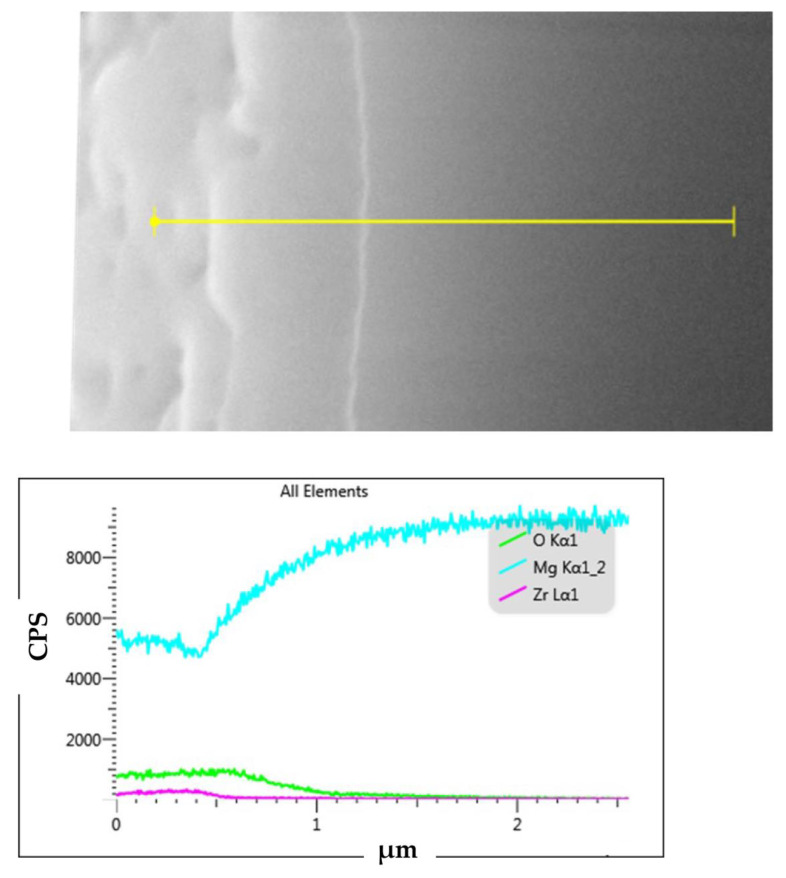
Cross-section observation and EDS depth profiles of O, Mg, and Zr for ZrO_2_-coated Mg specimen.

Figure 14 showcases FIB visuals for ZrO_2_-coated (a), CaP/ZrO_2_-coated (b), CaP-Hep/CaP/ZrO_2_-covered (c), and CaP-Gel-Hep/CaP-Hep/CaP/ZrO_2_-covered samples (d) in order. The ZrO_2_ layer has an approximate thickness of 0.86 μm, as depicted in Figure 15, which also provides the EDS depth analysis of the specimen coated with ZrO_2_. However, some mud cracks are visible on the ZrO_2_ coating.

As the second layer of CaP is coated on the ZrO_2_-coated specimens, the mud cracks can be occasionally covered by the CaP coating, and a porous grass-like HA layer of approximately 4.5 μm is formed.

For the third layer of CaP-Hep coating deposited on CaP/ZrO_2_, some porous regions are filled in, but mud cracks are still found and enlarged, as shown in Figure 14c. This is due to dehydration after drying, resulting in almost the same thickness as the CaP/ZrO_2_ coating. There is no clear boundary to distinguish the CaP-Hep layer from the CaP layer.

Conversely, the fourth layer of the CaP-Gel-Hep coating is more compact, thoroughly covering and sealing the surface of the specimen after coating, as demonstrated in Figure 14d. 

Furthermore, Figure 15 presents cross-section observations and EDS depth profiles of O, Mg, and Zr for ZrO_2_-coated specimens, indicating the complete mixing of Zr and Mg elements in the coating without spalling.

### 3.3. Corrosion Evaluation

**Figure 16:**
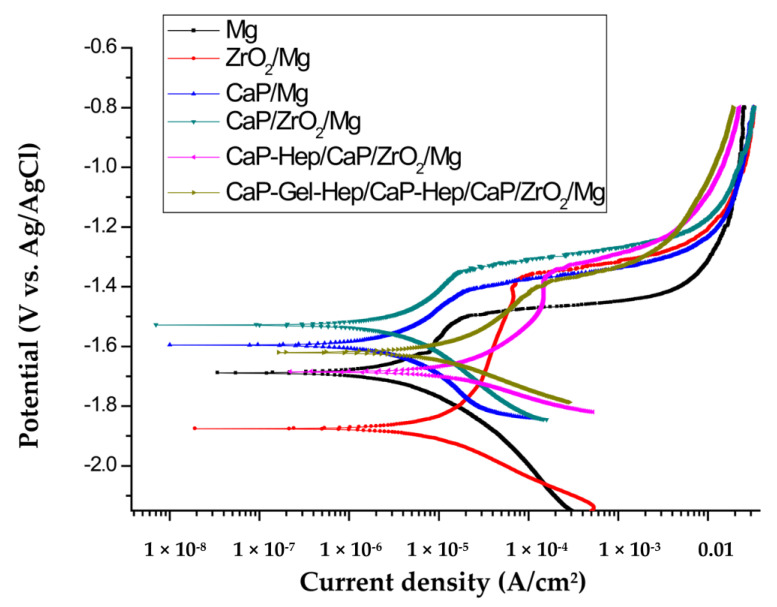
Potentiodynamic polarization graphs for CaP-Gel-Hep/CaP-Hep/CaP/ZrO_2_-coated, CaP-Hep/CaP/ZrO_2_-coated, CaP/ZrO_2_-coated, CaP-coated, ZrO_2_-coated, and bare Mg samples, all tested in MEM supplemented with 10% FBS at 37 °C.

Figure 16 showcases the potentiodynamic polarization graphs for six distinct samples: CaP-Gel-Hep/CaP-Hep/CaP/ZrO_2_-coated, CaP-Hep/CaP/ZrO_2_-coated, CaP/ZrO_2_-coated, CaP-coated, ZrO_2_-coated, and bare samples, each submerged in a simulated human physiological environment, i.e., a minimum essential medium (MEM) supplemented with 10% fetal bovine serum (FBS). The derived corrosion potentials (*E_corr_*), corrosion current densities (*I_corr_*), pitting potentials (*E_pit_*), and corrosion rate (CR) are listed in Table 4. The corrosion rate (CR) can be inferred from the *I_corr_* value as shown below [33].

(10) $$CR=K\frac{Icorr}{\rho }EW$$

The corrosion rate (CR) is expressed in mm/yr, while the corrosion current density (*I_corr_*) is measured in μA/cm^2^. The value of K is 3.27 × 10^−3^ mm g/μA cm yr. To calculate the equivalent weight (EW), the density of the substrate (ρ) in g/cm^3^ and the atomic fractions of each alloying element must be taken into consideration.

The corrosion current density (*I_corr_*) of the uncoated magnesium specimen was measured at 1.33 × 10^−5^ A/cm^2^, which is lower than that in 0.1 M NaCl solution [34]. This indicates that the corrosion behavior is influenced by the components present in the solution. Specifically, CO3^2−^, PO4^3−^, and OH^−^ ions promote the formation of a passivation film, while Cl^−^ ions tend to destroy it. As a result, the uncoated specimen exhibits only a small passivation region when exposed to artificial blood plasma.

In comparison, the *I_corr_* of the ZrO_2_-coated specimen was slightly reduced to 7.10 × 10^−6^ A/cm^2^. Despite the observation of mud cracks on the ZrO_2_-coated specimens in FESEM images, as shown in Figure 9a,b, the ZrO_2_ coating effectively inhibits the release of magnesium ions and delays the formation of Mg (OH)_2_ and magnesium phosphate films. This results in the appearance of a passivation region at a higher current density in the anodic curve.

The addition of the second layer of CaP coating not only covered the mud cracks of the ZrO_2_ coating but also increased the overall thickness of the specimen, leading to a significant reduction in *I_corr_* for the CaP/ZrO_2_-coated specimen to 3.86 × 10^−6^ A/cm^2^. Additionally, *E_corr_* was enhanced compared to the single-layer CaP coated specimen.

However, the third layer of CaP-Hep coating on the CaP/ZrO_2_ specimen resulted in an increase in *I_corr_* due to the enlargement of some cracks caused by the shrinkage of the CaP-Hep composite film during the drying process. Moreover, parts of the CaP-coated layers were dissolved during the third layer process, preventing a significant increase in film thickness. Consequently, *I_corr_* of the CaP-Hep/CaP/ZrO_2_-coated specimen increased to 1.04 × 10^−5^ A/cm^2^.

Conversely, the fourth layer of CaP-Gel-Hep applied to the CaP-Hep/CaP/ZrO_2_-coated sample resulted in more compact and substantial films, causing a decrease in *I_corr_* for the CaP-Gel-Hep/CaP-Hep/CaP/ZrO_2_-covered sample to 7.42 × 10^−6^ A/cm^2^.

### 3.4. Weight Increase and Medication Encapsulation

**Figure 17:**
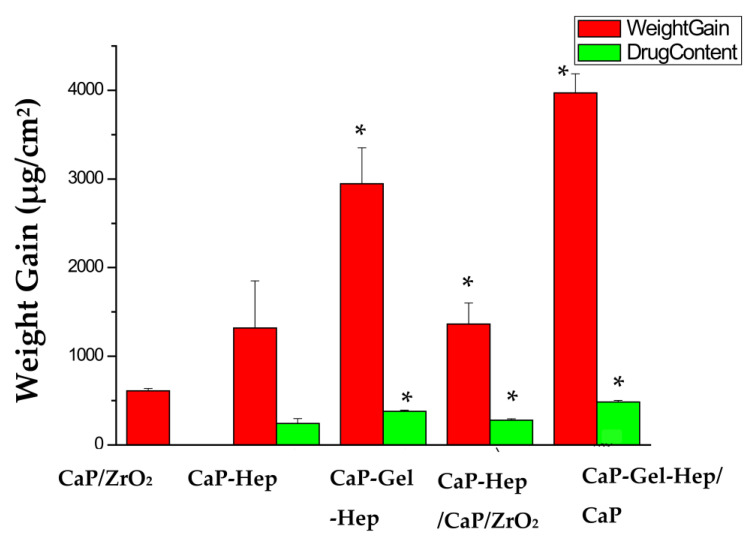
The increase in weight and drug amount in the CaP/ZrO_2_, CaP-Hep, CaP-Gel-Hep, CaP-Hep/CaP/ZrO_2_, and CaP-Gel-Hep/CaP-Hep/CaP/ZrO_2_ coatings. * Statistically significant difference between the control and coated groups at *p* < 0.05.

Figure 17 displays the weight gain and drug loading of the heparin-containing coating layers. The CaP-Gel-Hep coating exhibits a higher heparin content compared to the CaP-Hep coating. Heparin possesses highly negative charges due to its SO_3_^−^ and COO^−^ functional groups, making it challenging for electrochemical deposition on specimens due to strong repulsive forces among heparin molecules. To overcome this, heparin is mixed with a biopolymer in this study.

Heparin could combine with Ca^2+^ ions, forming heparin–calcium complex ions in the CaP solution. As previous research has shown successful deposition of hydroxyapatite on pure titanium specimens by electrochemical methods [31], in this case, the heparin–calcium complex ions react with HPO4^2−^, PO4^3−^, and OH^−^ to form the CaP-Hep composite coating during cathodic deposition on specimens. The CaP-Hep/CaP/ZrO_2_ multi-layer (277.87 ± 16.14 μg/cm^2^) contains more heparin than the CaP-Hep single layer (243.56 ± 55.18 μg/cm^2^). FESEM images of the CaP-Hep/CaP/ZrO_2_ composite show that calcium phosphate mixed with heparin (CaP-Hep) coating grows on porous grass-like HA, providing a larger surface area compared to bare magnesium.

The drug quantity in CaP-Gel-Hep exceeds that in CaP-Hep, likely due to the robust integration of heparin with gelatin prior to merging with calcium phosphate. Gelatin, like many proteins, is an amphoteric protein possessing an isoelectric point (pI or IEP) that ranges between 5 and 9, influenced by the source and production technique. The pH level of CaP-Gel-Hep stands at 4.85, below its IEP, which induces a positive charge in Gel from the protonation of amino (NH^3+^) groups. The NH^3+^ units in Gel can engage with the negatively charged heparin, and H^+^ can bind with the heparin’s carboxyl groups in the solution via the n COO^−^ + n H^+^ → n COOH reaction. During the cathodic deposition in the CaP-Gel-Hep mixture, heparin–calcium complex ions and the Gel-Hep blend react with HPO_4_^2−^, PO_4_^3−^, and OH^−^ to produce the CaP-Gel-Hep composite (379.05 ± 13.63 μg/cm^2^) This leads to enhanced drug encapsulation in comparison to CaP-Hep (243.56 ± 55.18 μg/cm^2^). Furthermore, the CaP-Gel-Hep/CaP-Hep/CaP/ZrO_2_ multi-layered coating has the highest heparin content (484.19 ± 19.26 μg/cm^2^). However, some heparin in the third-layer CaP-Hep might dissolve during the deposition process. Yet, the drug encapsulation in the CaP-Gel-Hep/CaP-Hep/CaP/ZrO_2_ multi-layer surpasses that in the CaP-Hep/CaP/ZrO_2_ multi-layer shown in Table 5.

### 3.5. Heparin Release In Vitro 

**Figure 18:**
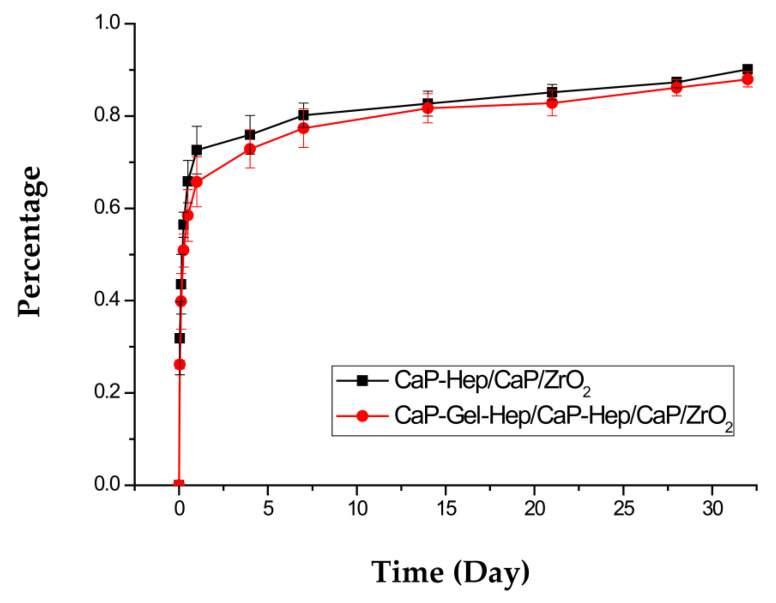
Heparin discharge patterns from Mg specimens coated with CaP-Hep/CaP/ZrO_2_ and CaP-Gel-Hep/CaP-Hep/CaP/ZrO_2_.

The patterns of heparin discharge from CaP-Hep/CaP/ZrO_2_- and CaP-Gel-Hep/CaP-Hep/CaP/ZrO_2_-covered pure magnesium samples in 4 mL PBS at 37 °C, depicted in Figure 18, can be segmented into three phases: a preliminary surge (day 1), controlled breakdown (days 2–7), and pore-driven diffusion (days 8–28).

For CaP-Hep/CaP/ZrO_2_-coated specimens, there is a significant initial burst release of heparin, accounting for about 70% of the total drug content on day 1. Similarly, CaP-Gel-Hep/CaP-Hep/CaP/ZrO_2_-covered samples show an initial burst release of approximately 65%. This difference may be attributed to the stronger combination between Gel and Hep compared to that between CaP and Hep. The initial burst release is essential as it can trigger vascular smooth muscle cell (VSMC) migration, necessary for healing blood vessel injuries, due to platelet-derived growth factor (PDGF) released by activated platelets [35]. This stage primarily results from heparin release from the composite surface layers containing CaP and Gel.

During the second stage, round rose-like particles composed of CaP-Gel-Hep gradually disappear, as shown in Figure 13, primarily due to the degradation of gelatin, leading to a cumulative heparin amount of up to 80%. FESEM images and X-ray patterns indicate the re-precipitation of HA on the specimen during immersion, resulting in a denser coating with smaller pores and randomly oriented HA after 7 days of immersion. Furthermore, sustained release is observed until 28 days, with a cumulative released heparin amount of up to 87%. It is theorized that the migration of heparin from the porous layer has a notable impact during this time.

### 3.6. MTT Test

**Figure 19:**
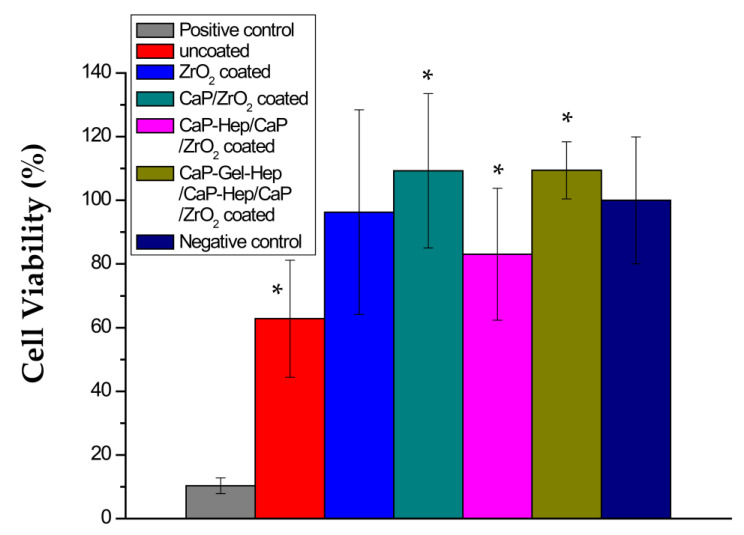
The cell viability of EA. hy926 cells was assessed after culturing with positive control, negative control, and extracts of media from uncoated, ZrO_2_-covered, CaP/ZrO_2_-covered, CaP-Hep/CaP/ZrO_2_-covered, and CaP-Gel-Hep/CaP-Hep/CaP/ZrO_2_-covered samples immersed for 1 day. * Statistically significant difference between control and coated groups at *p* < 0.05.

Figure 19 showcases cell survival rates in media extracted from the positive control, bare, ZrO_2_-coated, CaP/ZrO_2_-coated, CaP-Hep/CaP/ZrO_2_-coated, and CaP-Gel-Hep/CaP-Hep/CaP/ZrO_2_-covered samples after a day of incubation, in comparison to the negative control (just the medium). The survival rate of cells in ZrO_2_-coated samples surpasses that of the bare ones. Additionally, specimens coated with CaP/ZrO_2_ demonstrate superior cell survival compared to both ZrO_2_-coated and uncoated specimens. This observation suggests a possible relationship between cell viability and corrosion current density. In other words, the presence of a large quantity of Mg^2+^ ions released from Mg corrosion, resulting in a Mg concentration of 180.89 ppm, could be toxic to endothelial cells, thus impacting cell viability. The slightly reduced cell viability for CaP-Hep/CaP/ZrO_2_-coated specimens further supports this hypothesis, as its corrosion current density increases slightly. The same argument is applicable to CaP-Gel-Hep/CaP-Hep/CaP/ZrO_2_-covered samples, which exhibit higher viability and lower corrosion current density compared to CaP-Hep/CaP/ZrO_2_-coated specimens.

## 4. Summary and Conclusions

After immersing pure magnesium substrates in a 1 M NaOH solution, passivating films of Mg (OH)_2_ are prepared for subsequent coatings. These coatings include ZrO_2_, CaP/ZrO_2_, CaP-Hep/CaP/ZrO_2_, and CaP-Gel-Hep/CaP-Hep/CaP/ZrO_2_, and they were applied using the electrochemical method. The research draws several conclusions through various tests such as XRD, FESEM, FIB, dynamic polarization, drug loading and releasing, and MTT tests.

The CaP coatings on passivated and/or ZrO_2_-coated specimens contain a purer phase of HA compared to the untreated one. This is attributed to the former two having more OH bonds, which favor the formation of HA much more than other calcium phosphates such as DCPD and OCP. The amount of heparin in CaP-Hep composites, which manifest as extremely fine, needle-shaped powders, rises from 243.56 ± 55.18 μg/cm^2^ in CaP-Hep-coated samples to 277.87 ± 16.14 μg/cm^2^ in CaP-Hep/CaP/ZrO_2_-coated ones. This growth can be attributed to the greater surface coverage of the latter. Furthermore, the robust connection between gelatin and heparin amplifies drug encapsulation to 379.05 ± 13.63 μg/cm^2^ for the bead-like CaP-Gel-Hep coating layered on samples. This culminates in a total heparin amount of 484.19 ± 19.26 μg/cm^2^ for the CaP-Gel-Hep/CaP-Hep coating on previously CaP/ZrO_2_-coated samples.

To evaluate the biodegradation (corrosion resistance) of uncoated and coated specimens, potentiodynamic polarization tests and immersion tests were carried out in a simulated human physiological environment, i.e., a minimum essential medium (MEM) supplemented with 10% fetal bovine serum (FBS). The corrosion current density (*I _corr_.*) of unaltered magnesium decreases from 13.3 to 3.86 μA/cm^2^ with the application of the CaP/ZrO_2_ coating. However, some cracks are found on CaP-Hep coatings after the drying process, leading to a slight increase in *I_corr_* to 10.5 μA/cm^2^. The denser CaP-Gel-Hep coating further reduces *I_corr_* to 7.42 μA/cm^2^.

The immediate release of 100% heparin from CaP-Hep and CaP-Gel-Hep decreases to 70% in CaP-Hep/CaP/ZrO_2_-coated samples and drops further to 65% in CaP-Gel-Hep/CaP-Hep/CaP/ZrO_2_-coated samples on the first day. This decline is due to the more robust connection between Gel and Hep via electrostatic forces as compared to the bond between CaP and Hep through hydrogen bonds. The reformation of HA with a non-specific alignment can compact the porous framework during immersion evaluations, leading to a prolonged release governed by diffusion.

Human umbilical vein endothelial cells (EA. hy926 cells, ATCC CRL 2922) were cultured in Dulbecco’s modified Eagle’s medium (DMEM) supplemented with 10% fetal bovine serum (FBS). The cells were maintained in a 5% CO_2_ atmosphere at 37 °C for the MTT test and the cell viability in the medium extracted from various specimens is directly related to their corrosion resistance. An elevated concentration of Mg^2+^ ions (180.89 ppm), which are produced from untreated Mg, proves to be toxic to endothelial cells. In contrast, specimens coated with CaP-Gel-Hep/CaP-Hep/CaP/ZrO_2_, CaP/ZrO_2_, and ZrO_2_ demonstrate no harm to endothelial cells, indicating their potential for porous biodegradable stents or even scaffolds.

## Figures and Tables

**Table 1:** The composition details of magnesium.

Element	Mg	Al	Cu	Mn	Ni	Si	Fe
%	99.93	0.018	0.0033	0.019	0.0003	0.018	0.004

**Table 2:** pH levels, O_2_ levels, and conductivities of CaP-Gel-Hep solutions, CaP-Gel, CaP-Hep, CaP, Hep, and DI water.

Solution	pH Levels	O_2_ Levels (ppm)	Conductivity (mS/cm)
DI water	6.82	7.12	0.007
Hep	6.77	7.60	9.30
Calcium phosphate (CaP)	4.28	7.99	8.73
CaP-Hep	4.40	7.98	9.32
CaP-Gel	4.78	7.44	8.46
CaP-Gel-Hep	4.84	7.24	8.70

**Table 3:** Concentrations of inorganic salts and main organic components in plasma and MEM supplemented with 10% FBS.

Component	Plasma	MEM + FBS
Na^+^ (mmol/L)	142	151
K^+^ (mmol/L)	5.0	5.33
Ca^2+^ (mmol/L)	2.5	1.80
Mg^2+^ (mmol/L)	1.5	0.81
HCO_3_^−^ (mmol/L)	27.0	26.2
Cl^−^ (mmol/L)	103.0	125
HPO_4_^−^ (mmol/L)	1.0	1.01
SO_4_^2−^ (mmol/L)	0.5	0.81
Glucose (mmol/L)	3.6–5.2	5.56
Protein (g/L)	63–80	4.3
Amino acid (g/L)	ND	0.86
Phenol red (mmol/L)	-	0.03

ND: No data available.

**Table 4:** The outcomes from the potentiodynamic polarization tests on CaP-Gel-Hep/CaP-Hep/CaP/ZrO_2_-coated, CaP-Hep/CaP/ZrO_2_-coated, CaP/ZrO_2_-coated, CaP-coated, ZrO_2_-coated, and bare magnesium samples, all evaluated in MEM supplemented with 10% FBS at 37 °C, are showcased.

Tests *	A *	B *	C *	D *	E *
Specimens					
Pure magnesium	−1.69	1.33 × 10^−5^	−1.50	0.300	7.442
ZrO_2_ coated	−1.87	7.10 × 10^−6^	−1.38	0.160	3.973
CaP coated	−1.60	4.17 × 10^−6^	−1.41	0.094	2.333
CaP/ZrO_2_ coated	−1.53	3.86 × 10^−6^	−1.35	0.087	2.160
CaP-Hep/CaP/ZrO_2_ coated	−1.69	1.04 × 10^−5^	−1.37	0.235	5.820
CaP-Gel-Hep/CaP-Hep/CaP/ZrO_2_ coated	−1.62	7.42 × 10^−6^	−1.39	0.168	4.152

* Test **A**: Corrosion potential *E_corr_* (V vs. Ag/AgCl), **B**: Corrosion current density *I_corr_* (A/cm^2^), **C**: Pitting potential *E_pit_* (V vs. Ag/AgCl), **D**: Corrosion rate (mm/yr), **E**: Evaluated Mg^2+^ concentration (mmol/L).

**Table 5:** The medication encapsulation, weight increase, and drug content percentage for CaP/ZrO_2_-coated, CaP-Hep-coated, CaP-Gel-Hep-coated, CaP-Hep/CaP/ZrO_2_-coated, and CaP-Gel-Hep/CaP-Hep/CaP/ZrO_2_-coated Mg samples.

Items	Medication Encapsulation (μg/cm^2^)	Weight Increase (mg/cm^2^)	Drug Content/Weight Gain (×100%)
Specimens			
CaP/ZrO_2_		0.61 ± 0.03	
CaP-Hep	243.56 ± 55.18	1.32 ± 0.53	18.45%
CaP-Gel-Hep	379.05 ± 13.63 *	2.95 ± 0.4 *	12.84%
CaP-Hep/CaP/ZrO_2_	277.87 ± 16.14 *	1.36 ± 0.24 *	20.43%
CaP-Gel-Hep/CaP-Hep/CaP/ZrO_2_	484.19 ± 19.26 *	3.97 ± 0.22 *	12.20%

* Statistically significant difference between the control and coated groups at *p* < 0.05.

## Data Availability

The data presented in this study are available on request from the corresponding author. The data are not publicly available due to privacy.
